# Supplementary material for: A stepped-wedge randomised trial on the impact of early ART initiation on HIV-patients’ economic outcomes in Eswatini
Source: eLife. 2020 Aug 24;9:e58487. doi: 10.7554/eLife.58487 (PMC7529454; doi:10.7554/eLife.58487)
Supplement: Source data 1. [file elife-58487-data1.zip › Manuscript_Revised_Track Changes clean.docx]

**A Stepped-Wedge Randomised-Controlled Trial on the Impact of Early ART Initiation on HIV Patients’ Economic Welfare in Eswatini**

**Janina I Steinert**^§^, Technical University of Munich, Munich, Germany

**Shaukat Khan**, Clinton Health Acccess Initiative, Boston, US

**Khudzie Mlambo**, Clinton Health Acccess Initiative, Boston, US

**Fiona J Walsh**, Clinton Health Acccess Initiative, Boston, US

**Emma Mafara**, Clinton Health Acccess Initiative, Boston, US

**Charlotte Lejeune**, Clinton Health Acccess Initiative, Boston, US

**Cebele Wong**, Clinton Health Acccess Initiative, Boston, US

**Anita Hettema**, Clinton Health Acccess Initiative, Boston, US

**Osondu Ogbouji**, Duke Global Health Institute, Durham, US

**Sebastian Vollmer**, University of Göttingen, Göttingen, Germany

**Jan-Walter De Neve**, Heidelberg Institute of Global Health, Heidelberg University, Heidelberg, Germany

**Sikhathele Mazibuko**, Ministry of Health of the Kingdom of Eswatini, Mbabane, Eswatini

**Velephi Okello**, Ministry of Health of the Kingdom of Eswatini, Mbabane, Eswatini

**Till Bärnighausen**, Heidelberg Institute of Global Health, Heidelberg University, Heidelberg,

Germany

**Pascal Geldsetzer**, Division of Primary Care and Population Health, Department of Medicine,

Stanford University, Stanford, CA, United States & Heidelberg Institute of Global Health, Heidelberg University, Heidelberg, Germany

*^§^Corresponding Author*

[*janina.steinert@tum.de*](about:blank)

*Richard-Wagner Str. 1*

*80333 München*

*Germany*

# **Abstract**

***Background:*** Since 2015, the World Health Organisation (WHO) recommends immediate initiation of antiretroviral therapy (ART) for all HIV-positive patients. Epidemiological evidence points to important health benefits of immediate ART initiation; however, the policy’s economic impact remains unknown.

***Methods:*** We conducted a stepped-wedge cluster-randomised controlled trial in Eswatini to determine the causal impact of immediate ART initiation on patients’ economic welfare. Fourteen healthcare facilities were non-randomly matched in pairs and then randomly allocated to transition from the standard of care (ART eligibility at CD4 counts of < 350 cells/mm3 until September 2016 and <500 cells/mm3 thereafter) to the “Early Initiation of ART for All” (EAAA) intervention at one of seven timepoints. Patients, healthcare personnel, and outcome assessors remained unblinded. Data was collected via standardised paper-based surveys with HIV-positive, ART-naïve adults who were neither pregnant nor breastfeeding. Outcomes were patients’ time use, employment status, household expenditures and household wealth.

***Results:*** A total sample of 3,019 participants were interviewed over the duration of the study. The mean number of participants approached at each facility and time step varied from 4 to 112 participants. Using mixed-effects negative binomial regressions accounting for time trends and clustering, we found no significant difference between study arms for any economic outcome. Specifically, the EAAA intervention had no significant effect on non-resting time use (RR= 1.00, [CI: 0.96, 1.05, p=0.93]) or income-generating time use (RR= 0.94, [CI: 0.73,1.20, p=0.61]). Employment and household expenditures decreased slightly but not significantly in the EAAA group, with risk ratios of 0.93 [CI: 0.82, 1.04, p=0.21] and 0.92 [CI: 0.79, 1.06, p=0.26], respectively. We also found no significant treatment effect on households’ asset ownership and living standards (RR=0.96, [CI 0.92, 1.00, p=0.253]). Lastly, there was no evidence of heterogeneity in effect estimates by patients’ sex, age, education, timing of HIV diagnosis and ART initiation.

***Conclusions:*** Given the neutral effect on patients’ economic welfare but positive effects on health, our findings support further investments into scaling-up immediate ART for all HIV patients.

***Trial Registration:*** ClinicalTrials.gov, NCT02909218 and NCT03789448; ethical approval: Eswatini National Health Service Review Board & Harvard T.H. Chan School of Public Health Review Board.

# **Background**

Recent trials have pointed to substantial health benefits of immediate antiretroviral therapy (ART) initiation for all HIV-positive patients compared to initiating ART based on a CD4-cell count threshold. Benefits include reduced HIV-related mortality and morbidity and decreased transmission risk to HIV-negative sexual partners.^1–5^ In line with this epidemiological evidence, the World Health Organization (WHO) has updated its consolidated guidelines on the use of antiretrovirals in 2015, now advocating for immediate ART initiation (or “universal test and treat”) for all HIV-positive adults, adolescents, and children.^6^

In view of these major changes in ART provision, it is crucial for health policy makers to understand the implications of immediate ART initiation for HIV patients’ economic welfare. At high CD4-count levels, we would expect the majority of patients to be relatively healthy, and thus have productivity levels and out-of-pocket health expenditures that are similar to those of HIV-negative patients.^7^ While ART may still improve economic welfare among these patients through an improvement in health status, it may also decrease these patients’ economic welfare through, for example, the side effects of antiretroviral drugs, increased frequency of (ART) clinic visits or stigma from taking ART.^8,9^ The economic consequences of early ART initiation for this specific patient group are therefore unclear and have to date not been investigated experimentally.

Previous studies have assessed labour market outcomes and overall financial wellbeing of patients on ART, relative to patients not yet on ART. Of these, several studies have highlighted beneficial economic impacts of ART initiation, which are primarily based on the positive labour market effects of improved health. Accordingly, empirical evidence has pointed to higher work performance and productivity,^10–13^ lower absenteeism,^11^ increases in savings rates,^15^ as well as increased educational expenditures and attainment^15,16^ following ART initiation. Conversely, other studies have documented detrimental economic effects of ART initiation (even under universal access to ART schemes), largely driven by three suggested mechanisms: first, by increased patient-borne healthcare expenditures associated with travel to ART clinics, clinic and hospital fees, and income foregone;^17–19^ second, by elevated levels of food insecurity due to a treatment-induced increase in appetite and fewer financial resources to absorb the higher food expenditures;^20^ and third, by reduced productivity as a result of short-term adverse and toxic effects linked to antiretroviral drugs.^1,17^ However, these previous studies provide only little insights on the anticipated economic effects of immediate ART initiation because they are based on outdated treatment guidelines, thus comparing HIV-patients above and below a certain CD4 cell count level (e.g., 500 cells/mm^3^) that determines ART eligibility. Given that HIV-patients who are not yet on ART but have a relatively low CD4 count may be more susceptible to opportunistic infections and adverse events than those with higher CD4 counts, this comparison group is inadequate for assessing the economic consequences of the current WHO-endorsed ART initiation strategy that is independent of patients’ CD4 counts.

To decide whether and how much governments and international organisations should invest in scaling up immediate ART initiation for all HIV-patients, it is crucial to understand the impact of immediate ART initiation not only on health but also on HIV patients’ economic outcomes. This is the first randomised trial aimed at answering this question. Specifically, we conducted a stepped-wedge cluster-randomised controlled trial of the “Early Initiation of ART for All” (EAAA) intervention for HIV-patients in Eswatini to test the causal impact of immediate ART initiation on a range of economic outcomes, including patients’ time use, employment, household expenditures, and household living standards.

**Results**

## **Sample Characteristics**

Fourteen healthcare facilities (“clusters”) were consecutively enrolled into the Maximising ART for Better Health and Zero New HIV Infections (MaxART) stepped-wedge trial and 3,019 participants were interviewed over the duration of the study. The mean number of participants approached at each facility and time step varied from 3.5 to 112 participants (see Figure 2).

Table 2 summarises sociodemographic information separately for two study samples. The full sample was composed of 3019 participants, sampled across 14 healthcare facilities. Participants enrolled into the EAAA intervention arm were on average aged 38.3 years (range: 18-85 years), 71.0% were female, 53.5% were married, and 56.0% had completed at least some secondary schooling. Participants in the standard of care group had similar characteristics: 74.3% were female, 56.6% married, and 56.0% had completed at least some secondary education.

The random subset of participants with data on household expenditure and living standards was composed of 1485 patients who were also sampled across all 14 healthcare facilities. Overall, sociodemographic characteristics were very similar to those of the full sample.


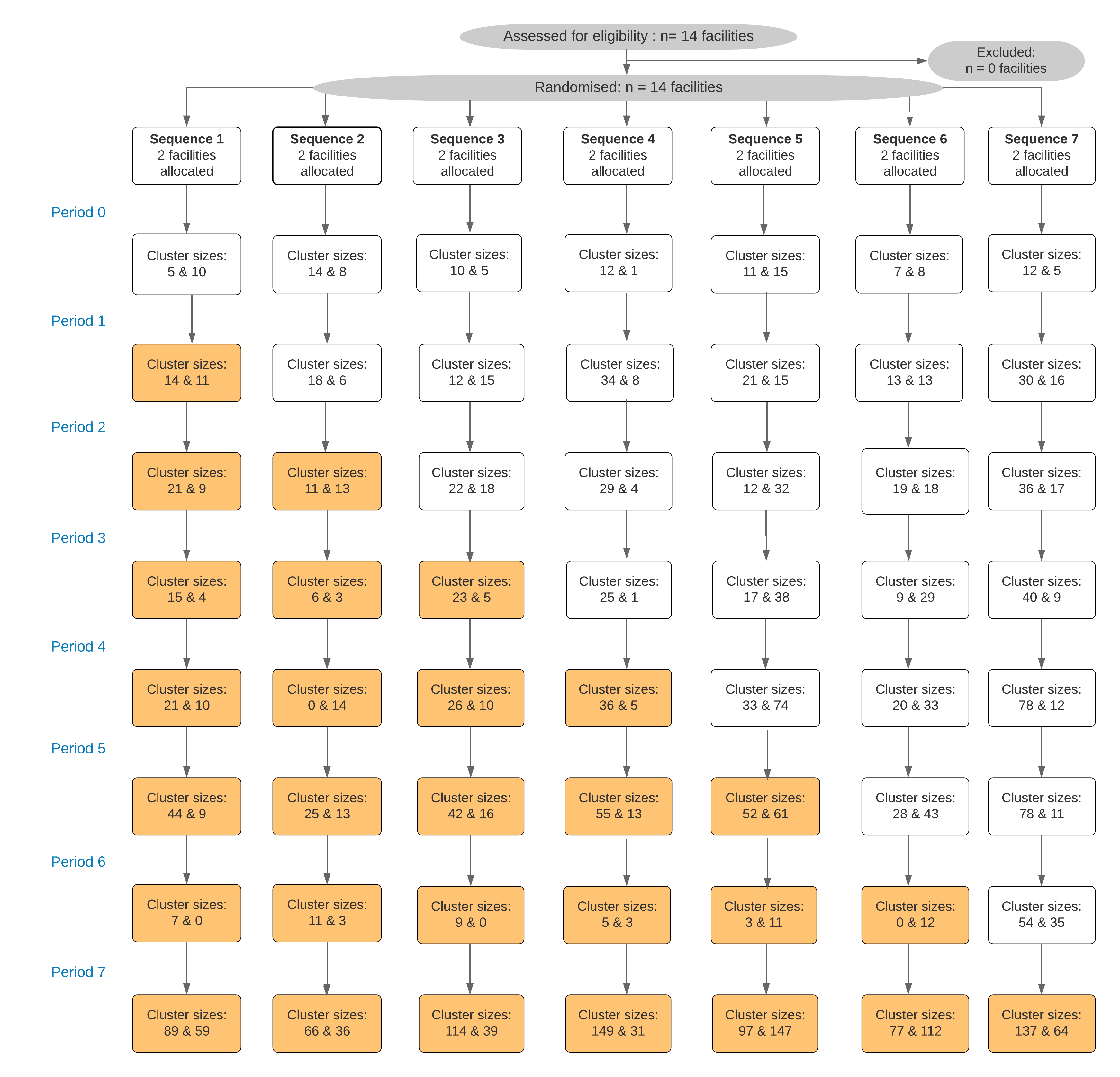
Figure 2. Participant Flow Chart (Full Sample)

Table 2. Sample characteristics

| **Full study sample (N=3019)** | | |
| --- | --- | --- |
|  | **EAAA**  **(N=1868)** | **SoC**  **(N=1151)** |
| Female, n (%) | 1326 (71.0%) | 855 (74.3%) |
| Age, mean (SD) | 38.3 (11.8) | 38.3 (11.8) |
| Education, n (%) |  |  |
| No formal schooling | 356 (19.1%) | 212 (18.6%) |
| Any primary schooling | 400 (21.4%) | 294 (25.5%) |
| Any secondary schooling | 1112 (59.5%) | 645 (56.0%) |
| Married, n (%) | 1000 (53.5%) | 651 (56.6%) |
| **Random subsample with data on household expenditure and living standards (N=1485)** | | |
|  | **EAAA**  **(N=930)** | **SoC**  **(N=555)** |
| Female, n (%) | 665 (71.5%) | 417 (75.1%) |
| Age, mean (SD) | 38.4 (11.9) | 38.2 (12.1) |
| Education, n (%) |  |  |
| No formal schooling | 175 (18.9%) | 99 (17.9%) |
| Any primary schooling | 192 (20.7%) | 142 (25.6%) |
| Any secondary schooling | 563 (60.5%) | 314 (56.6%) |
| Married, n (%) | 505 (54.3%) | 316 (56.9%) |
| Number of household members <15 years | 2.44 (1.11) | 2.58 (2.00) |
| Number of household members 15-60 years | 2.75 (2.24) | 3.21 (2.22) |
| Number of household members >60 years | 0.35 (0.61) | 0.45 (0.78) |

Notes: Abbreviations: EAAA, SD=standard deviation

## **EAAA Intervention Impact on Patient’s Economic Outcomes**

***Time Use***

The intervention impact on patient-level and household-level economic outcomes are presented in Figure 3. Histograms for all continuous outcome variables are presented in Figure 3 supplements 1-4. Participants in the EAAA group and in the standard of care group reported very similar levels of non-resting and income-generating time use. Non-resting time was approximately nine out of 24 hours in both study arms and the treatment effect was effectively null with an average marginal difference of only 0.6 minutes between groups (RR= 1.00, 95% CI: 0.96, 1.05, p=0.93). The treatment effect also remained precise and close to zero in alternative regression specifications, which included a random slope for time (see Supplementary File 1A). In addition, the results were similar when using a linear regression specification (β=0.02, 95% CI: -0.36, 0.39, p=0.93, see Supplementary File 1G). Income-generating time was also similar between both groups (RR= 0.94, 95% CI: 0.73,1.20, p=0.61), translating into an average marginal difference of only 12.6 minutes between patients in the EAAA phase and patients in the standard of care phase. The difference was not statistically significant and remained similarly small in alternative specifications (see Supplementary File 1B).

Figure 3. The Causal Effect of Early ART Initiation on Economic Outcomes


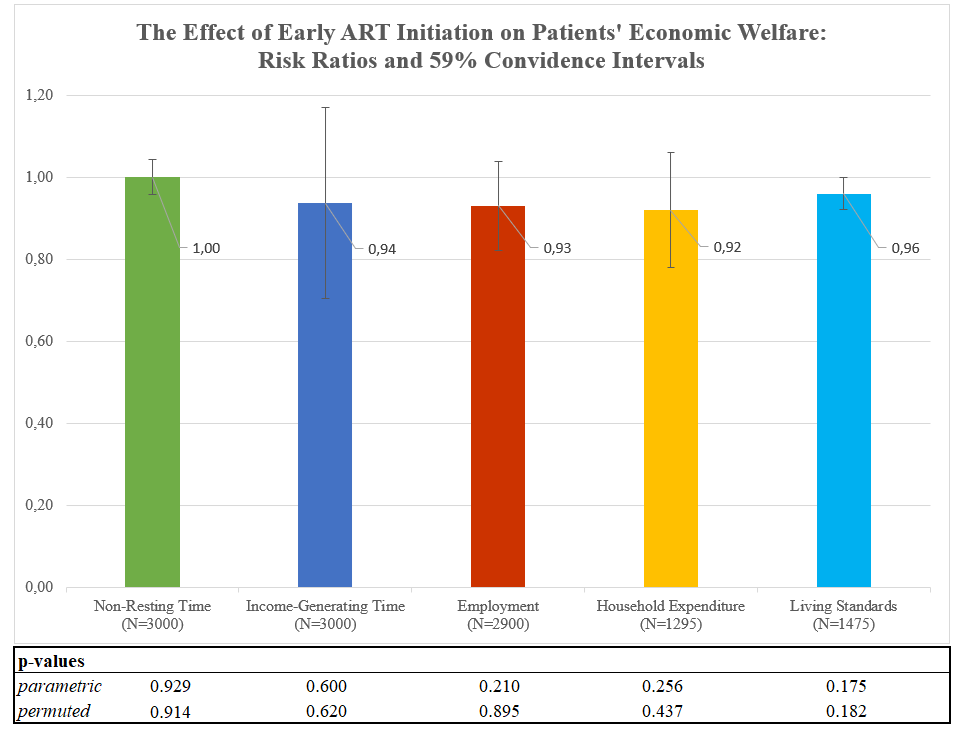


Notes: *Relative Risk presented for negative binomial mixed-effect regression with random intercept by healthcare facility (cluster) and a fixed effect for study period (Hussey & Hughes, 2007). All models control for respondent sex, age, marital status, and highest grade completed and were grand-mean centered. Parametric p-value obtained directly from the regression output; non-parametric p-value obtained from a permutation test with 1000 replications.*

***Employment***

We observed a decline in general employment rates over the entire study period, from 0.64 (SD=0.48) in study period 0 to 0.35 (SD=0.48) in study period 7 (see Figure 4). The employment trend observed in our study population stands in contrast to the national employment rate during the same period, which remained constant at 77-78%. The difference in employment status between study groups was small and statistically insignificant (RR=0.93, 95% CI: 0.82, 1.04, p=0.21, Figure 3). This finding remained robust across all alternative regression specifications (see Supplementary File 1C).

Figure 4. Average adjusted predictions of employment rates by period and study arm

##
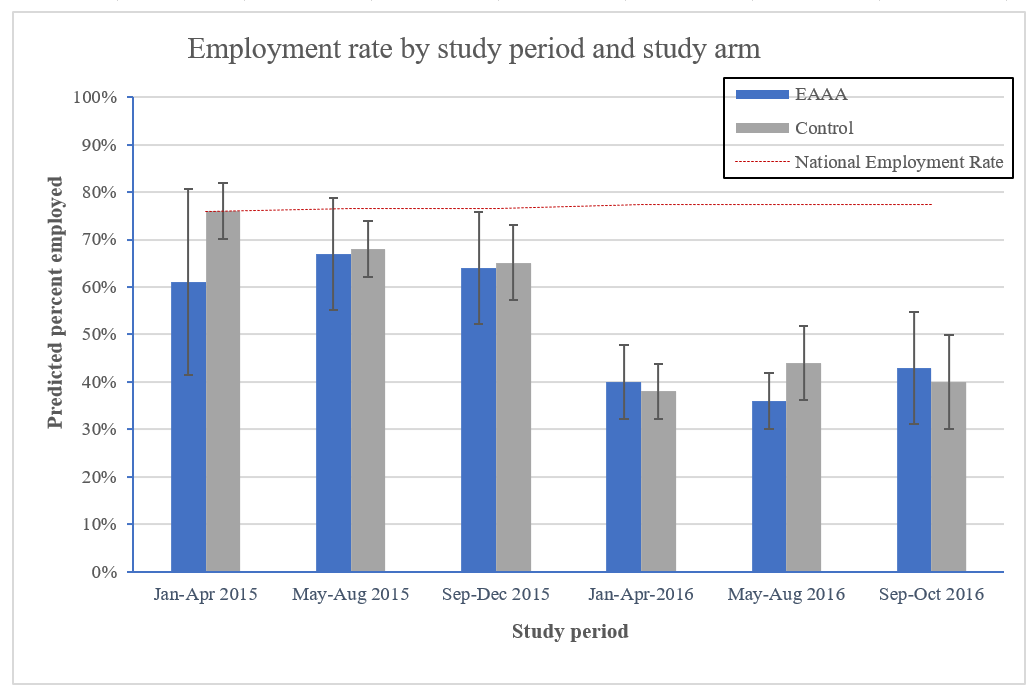
Notes: *Percent employed are the average adjusted predictions based on a logistic regression model with a time period fixed effect and a clinic-level random effect, interacting study period with trial arm, and controlling for patients’ age, sex, marital status, level of education, sex (binary), marital status (binary), and their level of education (continuous, specifying the highest grade completed). Period 0 and 7 are not shown because all participants interviewed in period 0 were part of the control phase and all participants interviewed in the last period were exposed to the intervention. The national total labour force participation rate is based on World Bank data and captures the proportion of the population of working age that is economically active during the reference period of one year.***EAAA Intervention Impact on Household-level Economic Outcomes**

***Household Expenditures***

Patients’ total past-month household expenditures were 10% lower in the EAAA intervention group but this difference was not statistically different from zero (RR=0.92, 95% CI: 0.79, 1.06, p=0.26, see Figure 3). All expenses were reported in Lilangeni (SZL) and converted into US dollars adjusted for purchasing power parity (PPP) and inflation for reporting purposes This corresponds to a reduction in the mean expected monthly expenses of 105.83 SZL (95% CI: -289.042 to 77.38498 SZL), or 20.47 PPP$ (95% CI: -55.93 $ to 14.97 $). Results remained virtually unchanged in alternative regression models (see Supplementary File 1D) or when imputing missing data using MICE (N= 1475) (see Supplementary File 1E).

***Household Living Standards***

Lastly, the EAAA intervention did not significantly affect patients’ living standards. From a total of 42 possible owned assets and housing quality indicators, counts of assets were very similar in both groups. Participants in the EAAA treatment group reported on average 0.71 indicators less than participants in the standard of care group (RR=0.96, 95% CI 0.92, 1.00, p=0.253). In alternative regression models (Supplementary File 1F), linear regressions (Supplementary Ffile 1G) and using an alternative principal-component-weighted outcome index (see Supplementary File 1H,), we found similar null effects.

## **Heterogeneity in Treatment Effects**

Overall, the causal random forests did not identify subgroups with effects that diverged significantly from the average treatment effect. Across outcomes, most heterogeneity was found along the variables (i) patients’ time on ART, (ii) number of months passed since patients’ HIV diagnosis, (iii) years of education completed, and (iv) age, whereas the importance metric for patients’ sex was very small, possibly due to an over-representation of women in our sample. The plots presented in Figure 3 supplements 5-9 depict heterogeneity in treatment effects along these four moderating variables. It appears that the program’s effect most economic welfare outcomes was slightly higher for patients with shorter rather than longer time on ART. However, it has to be cautioned that heterogeneity was not statistically significant for any of the four economic outcomes.

# **Discussion**

We present the first causal evaluation of the effect of immediate ART for all HIV patients on wider economic outcomes. Based on our primary results and several robustness checks, we are able to conclude that large harmful effects are very unlikely. More specifically, we found that neither patients’ time use nor their employment status and living standard were positively or negatively affected by the EAAA intervention. t. Although we found a reduction in monthly household expenditures among patients in the EAAA group, the magnitude was small in size (-126.17 SZL, corresponding to 3% of the average monthly household expenditures in Eswatini)^21^ and not statistically significant. Lastly, in machine-learning-supported heterogeneity analyses, we also did not find any patient subgroup for which the EAAA intervention either significantly improved or deteriorated overall economic welfare. Two previous publications based on the same trial have assessed how early ART initiation affected patients’ health, revealing a 6% higher retention in care rate in the EAAA group but no significant differences with regards to all-cause, disease-related, and HIV-related mortality between the EAAA and the standard of care group.^22,23^ While we were unable to link responses from this survey to patients’ clinical data, we may still infer that more substantial health impacts would have been necessary to significantly affect patients’ economic welfare. It is also possible that both the health and economic benefits of early ART initiation only materialise after a longer follow-up time, beyond the 36-months observation period covered in this trial.^24^ A potential alternative explanation for the absence of strong and beneficial treatment effects could relate to the broader socioeconomic conditions of the study region. Hence, if income generation opportunities are generally constrained due to given economic circumstances, HIV-patients may be unable to find work, irrespective of whether they are healthy or not. If patients’ health status does not substantially impact their earning potential, other welfare indicators such as household expenditures and living standards are also unlikely to change. However, we partly alleviate this problem by adopting a broad definition of employment by including informal and short-term work and should therefore be able to capture even small changes in participants’ income generation activities.

In contrast to several prior studies,^12–15^ our findings did not exhibit any substantial detrimental financial and economic consequences of ART initiation. At the very least, our results suggest that ART-related adverse events were not substantial enough to provoke significant drops in patients’ productivity levels.^25^ We therefore add important new empirical evidence from the perspective of patients’ economic welfare, which – given that EAAA does not appear to have large detrimental effects on patients’ economic welfare – support the 2015 WHO recommendation to offer immediate ART initiation to all HIV-patients.

Our study has several key strengths. First, we examined a comprehensive set of outcome variables and are thus able to gain nuanced insights into participants’ overall economic situation. Although the different outcomes are likely correlated, time use and employment are patient-level variables whereas expenditures and living standards are captured at the household level. The latter two variables could thus be differently affected by the EAAA intervention depending on whether the patient is the household’s main breadwinner or not. Household savings could have been another possible welfare-related aspect to assess. However, the general savings rate in Eswatini is low^26^ and savings are often mainly used to smooth consumption, and thus likely highly correlated with overall household expenditures. Second, we have collected very detailed information on patients’ time use. Time use is a measure that is presumably highly sensitive to potential short-term changes in economic productivity and, given that we asked about the previous 24 hours, less prone to measurement error or bias from a long recall period. In view of the precise null effects for the time use outcome, we can more confidently conclude that immediate ART initiation had no harmful effects on patients’ overall productivity levels. Third, and arguably most importantly, this is the first randomised study - and thus the first study to allow for causal inference under no untestable assumptions – of the impact of immediate ART initiation on indicators of patients’ economic welfare.

This study has six main limitations. First, biological data on patients’ CD4 count levels and viral load were not collected. It was thus not possible to assess whether the effects of EAAA on patients’ economic outcomes were different among those patients who had a CD4 count close to the treatment threshold at the time of ART initiation. Second, participant recruitment was implemented within healthcare facilities and it is therefore possible that patients who generally attend healthcare services more regularly and reliably are overrepresented in the study sample. Third, participants were not followed-up on longitudinally, which implies that for each individual, we either have a measurement of the pre- or the post-intervention phase (but never for both). Our effect estimates are based on the comparison of patients in the standard of care phase with patients in the EAAA phase, and would turn invalid if there was significant imbalance in baseline characteristics between these two groups. However, this is unlikely in view of the sufficiently large sample size and the random selection of interview dates for each facility. Fourth, data on household expenditures and household living standards were only collected from a random subsample of 50% of patients. Given the wide confidence interval of the effect estimates for household expenditures and household living standards, it is possible that we would have been able to find a significant effect for these two outcomes of a size that would still be meaningful to health policy makers if we had had a larger sample size. Fifth, data was based on patients’ self-report. Especially with regards to household expenditures and time use, this limitation is likely to have led to some degree of measurement error due to recall problems.^27,28^ In addition, while monthly expenses were summarised into 20 distinct expenditure categories to reduce interview length and cost, this may have led to further measurement imprecisions, for instance through adding up expenses for numerous individual food items into an overall category of “total shopping for food and groceries”. Yet, we expect that these measurement errors and reporting biases occurred – on average – to an equal degree in the EAAA and standard of care group and are therefore unlikely to systematically bias our point estimates of the causal intervention effect. Lastly, the employment rate in our study sample diverged from the national employment rate during the same period. This discrepancy could be explained by (i) the composition of our sample, which consisted of 75% female patients and is therefore not representative for the population as a whole, (ii) the temporal disaggregation into tertials, which might reflect some seasonal fluctuations in our data, and (iii) the lack of regional labour force data for the general population in the Hhohho region, rather than the aggregated national data that we have used as a reference.

This study provides the first causal evidence on the effect of immediate ART initiation on economic outcomes. EAAA is unlikely to have detectable, harmful economic repercussions for HIV patients in Eswatini. This is an important finding for health policy making in that it buttresses the WHO recommendation to discard eligibility thresholds for ART from the perspective of patients’ economic welfare – a perspective that is often ignored in the setting of clinical recommendations, yet important to those who are directly affected by these recommendations.^29,30^

**Materials and Methods**

The Maximising ART for Better Health and Zero New HIV Infections (MaxART) trial^31^ (NCT02909218) and the economic outcome analysis presented in here ([NCT03789448](about:blank)) were pre-registered on ClinicalTrials.gov.

## **Study setting**

The study was implemented in North-Western Eswatini (formerly “Swaziland”). 27.0% of the general population in Eswatini are HIV-positive; the highest HIV prevalence worldwide.^32^ The trial enrolled fourteen government-managed health facilities located in the Hhohho region (see Figure 1). At the study’s outset in 2014, all health facilities provided comprehensive HIV care and treatment according to the national adult HIV treatment guidelines effective at the time, thus initiating ART according to prescribed CD4 count levels. According to the *Annual HIV Program Report* of 2014,^33^ almost 60% of HIV-patients in the Hhohho region had been initiated on ART in the year prior to the trial roll-out.

Figure 1. Map of the healthcare facilities that participated in the study


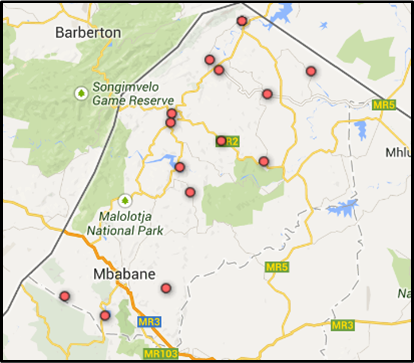


## **Stepped-wedge randomised trial design**

Health facilities were allocated non-randomly into seven pairs based on their geographic proximity to avoid possible contamination and based on their facility catchment size to ensure that group sizes were roughly equal. Over the course of three years, each of the seven pairs was randomly assigned to one of seven sequences, which determined the point in time at which each facility shifted from the standard of care (control condition) to the Early Access to ART for All (EAAA) intervention (treatment condition) (see Table 1). Hence, in the first period, all facilities adhered to the national standard of care while in the last period, all facilities had adopted EAAA. The randomisation was carried out by the trial statisticians. No stratification was used. This was an open-label trial in which healthcare providers and patients were unblinded to the intervention itself. However, the timing of the transition was only revealed to healthcare providers six to four weeks prior to the start of EAAA implementation.

Table 1. Stepped-wedge trial design used in this study

| **Healthcare Facility** | Sep - Dec  2014 | Jan – Apr 2015 | May - Aug 2015 | Sep-Dec 2015 | Jan -Apr 2016 | May-Aug 2016 | Sep -Oct 2016 | Oct 2016 -Aug 2017 |
| --- | --- | --- | --- | --- | --- | --- | --- | --- |
| Mshingishingini Nazarene Clinic | CONT | INT | INT | INT | INT | INT | INT | INT |
| Ntfonjeni Clinic | CONT | INT | INT | INT | INT | INT | INT | INT |
| Bulandzeni Clinic | CONT | CONT | INT | INT | INT | INT | INT | INT |
| Ndzingeni Clinic | CONT | CONT | INT | INT | INT | INT | INT | INT |
| Maguga Clinic | CONT | CONT | CONT | INT | INT | INT | INT | INT |
| Malandzela Clinic | CONT | CONT | CONT | INT | INT | INT | INT | INT |
| Pigg's Peak Hospital | CONT | CONT | CONT | CONT | INT | INT | INT | INT |
| Peak Nazarene Clinic | CONT | CONT | CONT | CONT | INT | INT | INT | INT |
| Herefords Clinic | CONT | CONT | CONT | CONT | CONT | INT | INT | INT |
| Ndvwabangeni Nazarene Clinic | CONT | CONT | CONT | CONT | CONT | INT | INT | INT |
| Sigangeni Clinic | CONT | CONT | CONT | CONT | CONT | CONT | INT | INT |
| Siphocosini Clinic | CONT | CONT | CONT | CONT | CONT | CONT | INTNT | INT |
| Horo Clinic | CONT | CONT | CONT | CONT | CONT | CONT | CONT | INT |
| Hhukwini Clinic | CONT | CONT | CONT | CONT | CONT | CONT | CONT | INT |

## **Control phase: standard of care**

In the standard of care phase, following national treatment guidelines effective at the time, ART eligibility was restricted to patients with CD4-cell counts of <350 cells/mm^3^ in the first 1.5 years of the study. In October 2016, the eligibility threshold was raised to CD4-cell counts <500 cells/mm^3^. Eligible patients were typically initiated on Eswatini’s first-line ART regimen (Tenofovir (TDF) + Lamivudine (3TC) + Efavirenz (EFV)). Those with contraindications to this regimen were initiated on alternative regimens, including TDF + 3TC + Nevirapine (NVP) or Zidovudine (AZT)  +  3TC + NVP (when EFV could not be used); Abacavir (ABC)  +  3TC + EFV or AZT + 3TC + EFV (when TDF could not be used); ABC + 3TC + EFV or Stavudine (D4T) +  3TC + EFV (when AZT could not be used). Patients attended one private and one group counselling session prior to initiation. While same-day ART initiation was allowed according to the national Integrated HIV Management Guidelines,^34^ HIV diagnosis and ART initiation in the respective facilities were typically a few days apart.

## **Intervention phase: Early Access to ART for All (EAAA)**

During the EAAA intervention phase, all patients who tested HIV-positive as well as patients enrolled in pre-ART care were offered immediate ART initiation, independent of their CD4-cell count. They received one counselling session and ART initiation on the same day and further monthly counselling after initiation. As in the standard of care, patients in the EAAA programme were initiated on Eswatini’s first-line treatment regimen or, if contraindicated, on the same alternative regimens detailed above.

## **Data collection**

Data was collected via standardised paper-based questionnaires over eight time periods (baseline and seven transitions). In every period, a sample of all HIV-care patients in each of the enrolled healthcare facilities was randomly selected. Eligibility was constrained to patients who were HIV-positive and ART-naïve, who were over the age of 18 years, and who were neither pregnant nor breastfeeding. Patients were eligible irrespective of whether ART initiation could take place on the same day of HIV diagnosis or a few days thereafter. For each facility, the study team randomly selected data collection days. On these days, the study team adopted the sampling strategy of selecting the next patient entering the consultation room. This strategy yields a representative sample if the sample size is sufficiently large and the order with which patients are seen by a clinician is random. Monte Carlo simulations have shown that this sampling strategy also tends to be more efficient and unbiased compared to simple and systematic random sampling, and does not underrepresent potentially healthier patients with shorter consultations as is the case when sampling those *exiting* the consultation room.^35^ Respondents gave verbal and written consent before completing the interview and were informed about their right to decline or withdraw their participation at any point in time. No prior sample size calculations were performed.

## **Outcomes**

We assessed the impact of the EAAA intervention on four economic outcomes. First, patients’ time use during the day prior to the interview was measured by collecting detailed information on hourly activities for a cycle of 24 hours. For our analysis, we specified two outcomes that are indicative of patients’ productivity levels: (i) “non-resting time” to capture the total hours spent on activities other than sleeping and resting, and (ii) “income-generating time” to capture the total hours spent on any income generation activities, which comprised formal employment, primary production activities in the informal sector, subsistence farming, and income generated from own businesses (i.e., from the sale of goods). The second outcome was patients’ current labour market participation, categorised as “employed” if patients were working or engaged in subsistence farming (either part- or full-time), and categorised as “not employed” if patients were unemployed, retired or taking sick or other leave. The third outcome was patients’ total past-month household expenditures on food- and non-food items, which was measured by asking each participant how much their household spends on 20 common expenditure items in a normal month (or, if the respondent preferred, in the past year) as well as on “other usual expenses” and “large purchases or expenses in the last 12 months” that were not mentioned in the list of common expenditure items. We opted for expenditure rather than income data because it is less affected by possible seasonal fluctuations in earnings and therefore better reflects a welfare level that households can maintain though consumption smoothing and informal borrowing.^36,37^ The last outcome was household living standards, measured as an additive index counting the total number of realised housing quality indicators (12 items, e.g., drinking water inside the house, concrete walls, flush toilet, etc.) and assets owned (30 items, e.g., refrigerator, phone, TV, animals, etc.). In line with economic literature,^27,36^ we also computed a principal-component-weighted index from the answers to these housing quality indicators and owned assets as an alternative metric to the additive index, reported in Supplementary File 1H Information on time use and employment was captured for the full sample. In order to reduce the length of the questionnaire, questions on household expenditures and household living standards were asked to every second participant who was interviewed.

## **Ethics**

Ethical approval for this study was obtained from the Eswatini National Health Service Review Board in July 2014 (Reference Number: MH/599C/FWA 000 15267). The study was further granted an exemption for non-human subjects research from the ethics review board of the Harvard T.H. Chan School of Public Health.

## **Data analysis**

We estimated the intent-to-treat effect (ITT) by comparing patients interviewed in the standard of care phase to patients interviewed in the EAAA phase (see EXHIBIT 2). We used mixed-effects negative binomial regressions (showing the resulting risk ratios) to account for the skewed distribution of some outcome variables (income-generating time and household expenditures). For normally distributed outcome variables (non-resting time and living standards), we additionally provide results from mixed-effects linear regressions in supplementary tables. For the binary employment outcome, we also estimated risk ratios for ease of interpretation by utilising a modified poisson regression model with a robust error structure.

Following the conventional Hussey and Hughes approach, regression models included a binary indicator (“fixed effect”) for each time period and a clinic-level random effect to account for clustering by clinic.^38^ While clinic-level random effects help to partly adjust for varying cluster size by assigning higher weights to larger clusters, we additionally included a permutation test to project more conservative p-values that correct for (i) the varying cluster sizes, (ii) the relatively small number of clusters, and (iii) potential violations in asymptotic properties of the regression models.^39^ Specifically, for each of the main outcome models (Hussey and Hughes model with control variables), we used a permutation test (implemented in the “swpermute” package in Stata^40^) with 1000 repetitions to test for the statistical significance of the treatment effect point estimates. In supplemental results, we present a second, more flexible, model that allows for potentially heterogeneous time trends across healthcare facilities by including a random slope for time period.^41^ Each of the two models was estimated without and with control variables, consisting of patients’ age (continuous), sex (binary), marital status (binary), and their level of education (continuous, specifying the highest grade completed). While adjustment for these variables is not needed to obtain unbiased effect estimates, their inclusion in the regressions might correct for small sample biases and improve precision.

180 participants (12% of the complete random subsample) did not respond to the household expenditure questions. For this outcome, we therefore ran two regression specifications, one based on the incomplete sample (i.e., a complete case analysis) and one based on a complete sample after imputing missing observations using multivariate imputation by chained equations (MICE).^42^ The imputation model was implemented using the *“mice”* package in Stata.^43^ We implemented the imputation with 1000 repetitions and included all variables used in the main outcome analysis as well as additional ‘auxiliary’ variables, which were current employment, education level, household living standards, and patients’ sociodemographic characteristics. Assuming that the likelihood of a missing value is only a function of observed characteristics, the MICE procedure iteratively estimates missing values based on Markov Chain Monte Carlo techniques. It creates 1000 complete datasets to estimate missing values, which are then averaged across all datasets.^44,45^

Lastly, we estimated for each of the five outcomes whether there was heterogeneity in treatment effects between different groups of patients. For this purpose, we utilised a machine learning approach in the form of a non-parametric causal forest algorithm.^46–48^ This approach has advantages over other subgroup tests^49,50^ in that it (i) does not require an a priori hypothesis on the potential differential effects, (ii) increases statistical power, (iii) and yields treatment effect estimates that are asymptotically normal.^47,48^ In this analysis, we first assess whether treatment effects for any subgroup are significantly different from the average treatment effect. In a second step, we explored the nature of potential heterogeneity through ordering moderating variables by their importance.

The random forest heterogeneity analysis was implemented in R 3.6.2. All other analyses were conducted in Stata 15.

**Competing Interests:** The authors declare no competing interests. The funder had no role in study design, data collection, data analysis, data interpretation, writing of the report or decision to publish. JS had full access to all the data in the study.

# **References**

1. The TEMPRANO ANRS 12136 Study Group. A Trial of Early Antiretrovirals and Isoniazid Preventive Therapy in Africa. *N Engl J Med*. 2015;373(9):808-822. doi:10.1056/NEJMoa1507198

2. Cohen MS, Chen YQ, McCauley M, et al. Antiretroviral Therapy for the Prevention of HIV-1 Transmission. *N Engl J Med*. 2016;375(9):830-839. doi:10.1056/NEJMoa1600693

3. The INSIGHT START Study Group. Initiation of Antiretroviral Therapy in Early Asymptomatic HIV Infection. *N Engl J Med*. 2015;373(9):795-807. doi:10.1056/NEJMoa1506816

4. Hayes RJ, Donnell D, Floyd S, et al. Effect of Universal Testing and Treatment on HIV Incidence — HPTN 071 (PopART). *N Engl J Med*. 2019;381(3):207-218. doi:10.1056/NEJMoa1814556

5. Ford N, Migone C, Calmy A, et al. Benefits and risks of rapid initiation of antiretroviral therapy. *AIDS Lond Engl*. 2018;32(1):17-23. doi:10.1097/QAD.0000000000001671

6. WHO | Consolidated guidelines on the use of antiretroviral drugs for treating and preventing HIV infection. WHO. Accessed April 1, 2019. http://www.who.int/hiv/pub/arv/arv-2016/en/

7. THIRUMURTHY H, CHAMIE G, JAIN V, et al. Improved employment and education outcomes in households of HIV-infected adults with high CD4 counts: evidence from a community health campaign in Uganda. *AIDS Lond Engl*. 2013;27(4). doi:10.1097/QAD.0b013e32835c54d8

8. DiBonaventura M daCosta, Gupta S, Cho M, Mrus J. The association of HIV/AIDS treatment side effects with health status, work productivity, and resource use. *AIDS Care*. 2012;24(6):744-755. doi:10.1080/09540121.2011.630363

9. Unge C, Johansson A, Zachariah R, Some D, Engelgem IV, Ekstrom AM. Reasons for unsatisfactory acceptance of antiretroviral treatment in the urban Kibera slum, Kenya. *AIDS Care*. 2008;20(2):146-149. doi:10.1080/09540120701513677

10. Bor J, Tanser F, Newell M-L, Bärnighausen T. Nearly Full Employment Recovery Among South African HIV Patients On Antiretroviral Therapy: Evidence From A Large Population Cohort. *Health Aff Proj Hope*. 2012;31(7). doi:10.1377/hlthaff.2012.0407

11. Larson BA, Fox MP, Rosen S, et al. Early effects of antiretroviral therapy on work performance: preliminary results from a cohort study of Kenyan agricultural workers. *AIDS Lond Engl*. 2008;22(3):421-425. doi:10.1097/QAD.0b013e3282f3cc0c

12. Beard J, Feeley F, Rosen S. Economic and quality of life outcomes of antiretroviral therapy for HIV/AIDS in developing countries: a systematic literature review. *AIDS Care*. 2009;21(11):1343-1356. doi:10.1080/09540120902889926

13. Thirumurthy H, Zivin JG, Goldstein M. The Economic Impact of AIDS Treatment Labor Supply in Western Kenya. *J Hum Resour*. 2008;43(3):511-552. doi:10.3368/jhr.43.3.511

14. Habyarimana J, Mbakile B, Pop-Eleches C. The impact of HIV/AIDS and ARV treatment on worker absenteeism: Implications for African firms. *J Hum Resour*. 2010;45(4):809-839. doi:10.3368/jhr.45.4.809

15. Baranov V, Kohler H-P. The Impact of AIDS Treatment on Savings and Human Capital Investment in Malawi. *Am Econ J Appl Econ*. 2018;10(1):266-306. doi:10.1257/app.20150369

16. Lucas AM, Chidothe M, Wilson NL. Effects of adult health interventions at scale on children’s schooling: Evidence from antiretroviral therapy in Zambia. *Econ Educ Rev*. 2019;72:107-120. doi:10.1016/j.econedurev.2019.04.006

17. Rosen S, Ketlhapile M, Sanne I, DeSilva MB. Cost to patients of obtaining treatment for HIV/AIDS in South Africa. *South Afr Med J Suid-Afr Tydskr Vir Geneeskd*. 2007;97(7):524-529.

18. Chimbindi N, Bor J, Newell M-L, et al. Time and money: the true costs of health care utilization for patients receiving ‘free’ HIV/TB care and treatment in rural KwaZulu-Natal. *J Acquir Immune Defic Syndr 1999*. 2015;70(2):e52-e60. doi:10.1097/QAI.0000000000000728

19. Leive A, Xu K. Coping with out-of-pocket health payments: empirical evidence from 15 African countries. *Bull World Health Organ*. 2008;86(11):849-856. doi:10.2471/BLT.07.049403

20. Patenaude BN, Chimbindi N, Pillay D, Bärnighausen T. The impact of ART initiation on household food security over time. *Soc Sci Med 1982*. 2018;198:175-184. doi:10.1016/j.socscimed.2017.11.036

21. The World Bank. Eswatini Household Income and Expenditure Survey- 2016/17 Final Report. Published online 2019.

22. Chao A, Spiegelman D, Khan S, et al. Mortality under early access to antiretroviral therapy vs. Eswatini’s national standard of care: the MaxART clustered randomized stepped-wedge trial. *HIV Med*. 2020;21(7):429-440. doi:10.1111/hiv.12876

23. Perriat D, Balzer L, Hayes R, et al. Comparative assessment of five trials of universal HIV testing and treatment in sub-Saharan Africa. *J Int AIDS Soc*. 2018;21(1). doi:10.1002/jia2.25048

24. May MT, Vehreschild J-J, Trickey A, et al. Mortality According to CD4 Count at Start of Combination Antiretroviral Therapy Among HIV-infected Patients Followed for up to 15 Years After Start of Treatment: Collaborative Cohort Study. *Clin Infect Dis*. 2016;62(12):1571-1577. doi:10.1093/cid/ciw183

25. Nansseu JRN, Bigna JJR. Antiretroviral therapy related adverse effects: Can sub-Saharan Africa cope with the new “test and treat” policy of the World Health Organization? *Infect Dis Poverty*. 2017;6(1):24. doi:10.1186/s40249-017-0240-3

26. World Bank Group. The Global Findex Database Measuring Financial Inclusion and the Fintech Revolution 2017. Published online 2017. https://globalfindex.worldbank.org/

27. Filmer D, Pritchett LH. Estimating Wealth Effects without Expenditure Data-or Tears: An Application to Educational Enrollments in States of India. *Demography*. 2001;38(1):115-132. doi:10.2307/3088292

28. Sahn DE, Stifel DC. Poverty Comparisons Over Time and Across Countries in Africa. *World Dev*. 2000;28(12):2123-2155. doi:10.1016/S0305-750X(00)00075-9

29. Govindasamy D, Ford N, Kranzer K. Risk factors, barriers and facilitators for linkage to antiretroviral therapy care: a systematic review. *AIDS*. 2012;26(16):2059–2067. doi:10.1097/QAD.0b013e3283578b9b

30. Fox MP, Mazimba A, Seidenberg P, Crooks D, Sikateyo B, Rosen S. Barriers to initiation of antiretroviral treatment in rural and urban areas of Zambia: a cross-sectional study of cost, stigma, and perceptions about ART. *J Int AIDS Soc*. 2010;13(1):8. doi:10.1186/1758-2652-13-8

31. Walsh FJ, Bärnighausen T, Delva W, et al. Impact of early initiation versus national standard of care of antiretroviral therapy in Swaziland’s public sector health system: study protocol for a stepped-wedge randomized trial. *Trials*. 2017;18(1):383. doi:10.1186/s13063-017-2128-8

32. Government of the Kingdom of Eswatini. Swaziland HIV Incidence Measurement Survey 2: A population-based HIV impact assessment. *Mbabane ICAP 2017*. Published online 2017.

33. Kingdom of Swaziland, Ministry of Health. Annual HIV Program Report - HTC, PMTCT, and ART. Published online 2014.

34. Ministry of Health, Kingdom of Swaziland. Swaziland Integrated HIV Managment Guidelines. Published online 2015. https://hivstar.lshtm.ac.uk/files/2017/11/Swaziland-integrated-HIV-management-guidelines-2015.pdf

35. Geldsetzer P, Fink G, Vaikath M, Bärnighausen T. Sampling for Patient Exit Interviews: Assessment of Methods Using Mathematical Derivation and Computer Simulations. *Health Serv Res*. 2018;53(1):256-272. doi:10.1111/1475-6773.12611

36. Sahn DE, Stifel D. Exploring Alternative Measures of Welfare in the Absence of Expenditure Data. *Rev Income Wealth*. 2003;49(4):463-489. doi:10.1111/j.0034-6586.2003.00100.x

37. Filmer D, Pritchett LH. Estimating Wealth Effects without Expenditure Data-or Tears: An Application to Educational Enrollments in States of India. *Demography*. 2001;38(1):115-132. doi:10.2307/3088292

38. Hussey MA, Hughes JP. Design and analysis of stepped wedge cluster randomized trials. *Contemp Clin Trials*. 2007;28(2):182-191. doi:10.1016/j.cct.2006.05.007

39. Athey S, Imbens G. The Econometrics of Randomized Experiments. *ArXiv160700698 Econ Stat*. Published online July 3, 2016. Accessed October 30, 2019. http://arxiv.org/abs/1607.00698

40. Thompson J. *SWPERMUTE: Stata Module to Compute Permutation Tests for Stepped-Wedge Cluster-Randomised Trials*. Boston College Department of Economics; 2019. Accessed October 30, 2019. https://ideas.repec.org/c/boc/bocode/s458426.html

41. Thompson JA, Fielding KL, Davey C, Aiken AM, Hargreaves JR, Hayes RJ. Bias and inference from misspecified mixed‐effect models in stepped wedge trial analysis. *Stat Med*. 2017;36(23):3670-3682. doi:10.1002/sim.7348

42. Azur MJ, Stuart EA, Frangakis C, Leaf PJ. Multiple Imputation by Chained Equations: What is it and how does it work? *Int J Methods Psychiatr Res*. 2011;20(1):40-49. doi:10.1002/mpr.329

43. Royston P, White IR. Multiple Imputation by Chained Equations (MICE): Implementation in Stata. *Journal of Statistical Software*.

44. Yu L-M, Burton A, Rivero-Arias O. Evaluation of software for multiple imputation of semi-continuous data. *Stat Methods Med Res*. 2007;16(3):243-258. doi:10.1177/0962280206074464

45. Azur MJ, Stuart EA, Frangakis C, Leaf PJ. Multiple Imputation by Chained Equations: What is it and how does it work? *Int J Methods Psychiatr Res*. 2011;20(1):40-49. doi:10.1002/mpr.329

46. Athey S, Tibshirani J, Wager S. Generalized random forests. *Ann Stat*. 2019;47(2):1148-1178. doi:10.1214/18-AOS1709

47. Athey S, Wager S. Estimating Treatment Effects with Causal Forests: An Application. *ArXiv190207409 Stat*. Published online February 20, 2019. Accessed February 21, 2020. http://arxiv.org/abs/1902.07409

48. Wager S, Athey S. Estimation and Inference of Heterogeneous Treatment Effects using Random Forests. *J Am Stat Assoc*. 2018;113(523):1228-1242. doi:10.1080/01621459.2017.1319839

49. Lee M. Non-parametric tests for distributional treatment effect for randomly censored responses. *J R Stat Soc Ser B Stat Methodol*. 2009;71(1):243-264. doi:10.1111/j.1467-9868.2008.00683.x

50. Crump RK, Hotz VJ, Imbens GW, Mitnik OA. Nonparametric Tests for Treatment Effect Heterogeneity. *Rev Econ Stat*. 2008;90(3):389-405.

# **Supplementary Documentation**

Supplementary File 1A. The causal effect of EAAA on non-resting time

Supplementary File 1B. The causal effect of EAAA on income-generating time

Supplementary File 1C. The causal effect of EAAA on employment

Supplementary File 1D. The causal effect of EAAA on household expenditures (non-imputed sample)

Supplementary File 1E. The causal effect of EAAA on household expenditures: Imputed sample

Supplementary File 1F. The causal effect of EAAA on asset and living standard index

Supplementary File 1G. OLS Specifications

Supplementary File 1H. The causal effect of EAAA on a principal component weighted asset and living standard index

Figure 3 – figure supplement 1. Histogram: Non-resting Time Use

Figure 3 – figure supplement 2Histogram: Income-Generating Time Use

Figure 3 – figure supplement 3. Histogram: Household Expenditures

Figure 3 – figure supplement 4: Household Assets/Living Standards

Figure 3 – figure supplement 5. Heterogeneity Plots for Non-Resting Time Use

Figure 3 – figure supplement 6. Heterogeneity Plots for Income-generating Time

Figure 3 – figure supplement 7. Heterogeneity Plots for Employment

Figure 3 – figure supplement 8. Heterogeneity Plots for Household Expenditures:

Figure 3 – figure supplement 9. Heterogeneity Plots for Household Assets

Source Data – Datasets, Dofiles, and R Code for Replication Purposes
