# Supplementary material for: A stepped-wedge randomised trial on the impact of early ART initiation on HIV-patients’ economic outcomes in Eswatini
Source: eLife. 2020 Aug 24;9:e58487. doi: 10.7554/eLife.58487 (PMC7529454; doi:10.7554/eLife.58487)
Supplement: Supplementary file 1. — (A) The causal effect of EAAA on non-resting time. (B) The causal effect of EAAA on income-generating time. (C) The causal effect of EAAA on employment. (D) The causal effect of EAAA on household expenditures (non-imputed sample). (E) The causal effect of EAAA on household expenditures: Imputed sample. (F) The causal effect of EAAA on asset and living standard index. (G) OLS Specifications. (H) The causal effect of EAAA on a principal component weighted asset and living standard index. [file elife-58487-supp1.docx]

**Supplementary files**

Supplementary File 1A. The causal effect of EAAA on non-resting time

|  | **Model 1^1^** | | **Model 2^3^** | | **Model 3^4^** | |
| --- | --- | --- | --- | --- | --- | --- |
|  | RR  [95% CI] | p-value | RR  [95% CI] | p-value | RR  [95% CI] | p-value |
| ***EAAA*** | **0.99**  **[-0.91-1.09]** | **0.933** | **1.00**  **[0.96, 1.04]** | **0.932** | **1.00**  **[0.96-1.05]** | **0.882** |
| *Female* | / |  | / |  | 1.07***  [1.04-1.11] | <0.001 |
| *Age* | / |  | / |  | 1.00  [0.99-1.00] | 0.056 |
| *Married* | / |  | / |  | 1.04***  [1.01-1.07] | 0.006 |
| *Education Status* | / |  | / |  | 1.01  [1.00-1.01] | 0.007 |
| N | 3019 |  | 3019 |  | 3000 |  |

^1^Mixed-effect regression with random intercept by healthcare facility (cluster) and a fixed effect for study period, thus assuming a homogeneous secular trend across clusters

^2^ Mixed-effect regression with random intercept by healthcare facility (cluster) and a random slope for study period, thus allowing for varying secular trends across clusters.

^3^ Same as *Model 2* but with additional control variables, including *sex, age, marital status, and education*. All control variables were grand-mean centered.

Supplementary File 1B. The causal effect of EAAA on income-generating time

|  | **Model 1^1^** | | **Model 2^2^** | | **Model 3^3^** | |
| --- | --- | --- | --- | --- | --- | --- |
|  | RR  [95% CI] | p-value | RR  [95% CI] | p-value | RR  [95% CI] | p-value |
| ***EAAA*** | **0.98**  **[0.76-1.26]** | **0.871** | **0.98**  **[0.76-1.25]** | **0.852** | **0.93**  **[0.73-1.19]** | **0.559** |
| *Female* | / |  | / |  | 0.49***  [0.42-0.58] | <0.001 |
| *Age* | / |  | / |  | 1.02***  (1.01-1.02] | <0.001 |
| *Married* | / |  | / |  | 0.99  [0.85-1.16] | 0.913 |
| *Education Status* | / |  | / |  | 1.00  [0.98-1.03] | 0.647 |
| N | 3019 |  | 3019 |  | 3000 |  |

RR= Risk Ratios from negative binomial regression

^1^Mixed-effect regression with random intercept by healthcare facility (cluster) and a fixed effect for study period, thus assuming a homogeneous secular trend across clusters

^2^ Mixed-effect regression with random intercept by healthcare facility (cluster) and a random slope for study period, thus allowing for varying secular trends across clusters.

^3^ Same as *Model 2* but with additional control variables, including *sex, age, marital status, and education*. All control variables were grand-mean centered.

Supplementary File 1C. The causal effect of EAAA on employment

|  | **Model 1^1^** | | **Model 2^2^** | | **Model 3^3^** | |
| --- | --- | --- | --- | --- | --- | --- |
|  | RR  [95% CI] | p-value | OR  [95% CI] | p-value | OR  [95% CI] | p-value |
| ***EAAA*** | **0.94**  **[0.84, 1.06]** | **0.308** | **0.95**  **[0.84, 1.07]** | **0.373** | **0.93**  **[0.83, 1.04]** | **0.199** |
| *Female* | **/** |  | **/** |  | 0.64***  [0.58, 0.71] | <0.001 |
| *Age* | **/** |  | **/** |  | 1.00***  [1.00, 1.01] | 0.006 |
| *Married* | **/** |  | **/** |  | 1.01  [0.96, 1.07] | 0.613 |
| *Education Status* | **/** |  | **/** |  | 1.02***  [1.01, 1.03] | <0.001 |
| N | 3000 |  | 3000 |  | 2990 |  |

RR= Risk Ratios from modified poisson regression model with a robust error variance

^1^Mixed-effect regression with random intercept by healthcare facility (cluster) and a fixed effect for study period, thus assuming a homogeneous secular trend across clusters

^2^ Mixed-effect regression with random intercept by healthcare facility (cluster) and a random slope for study period, thus allowing for varying secular trends across clusters.

^3^ Same as *Model 2* but with additional control variables, including *sex, age, marital status, and education*. All control variables were grand-mean centered.

Supplementary File 1D. The causal effect of EAAA on household expenditures (non-imputed sample)

|  | **Model 1^1^** | | **Model 2^2^** | | **Model 3^3^** | |
| --- | --- | --- | --- | --- | --- | --- |
|  | RR  [95% CI] | p-value | RR  [95% CI] | p-value | RR  [95% CI] | p-value |
| ***EAAA*** | **0.92**  **[0.79, 1.07]** | **0.286** | **0.92**  **[0.79, 1.08]** | **0.310** | **0.90**  **[0.77, 1.06]** | **0.212** |
| *Female* | / |  | / |  | 0.84***  [0.76, 0.94] | 0.001 |
| *Age* | / |  | / |  | 0.99***  [0.99, 1.00] | <0.001 |
| *Married* | / |  | / |  | 1.21***  [1.10, 1.33] | <0.001 |
| *Education Status* | / |  | / |  | 1.04***  [1.03, 1.06] | <0.001 |
| N | 1300 |  | 1300 |  | 1295 |  |

^1^Mixed-effect regression with random intercept by healthcare facility (cluster) and a fixed effect for study period, thus assuming a homogeneous secular trend across clusters

^2^ Mixed-effect regression with random intercept by healthcare facility (cluster) and a random slope for study period, thus allowing for varying secular trends across clusters.

^3^ Same as *Model 2* but with additional control variables, including *sex, age, marital status, and education*. All control variables were grand-mean centered.

Supplementary File 1E. The causal effect of EAAA on household expenditures: Imputed sample

|  | **Main Model** | |
| --- | --- | --- |
|  | β  [95% CI] | p_par_ |
| ***EAAA*** | 0.91  [0.78, 1.04] | 0.167 |
| N | **1475 (Imputed)** |  |

*Notes: The estimation is based on the Hussey & Hughes model with control variables.*

Supplementary File 1F. The causal effect of EAAA on asset and living standard index

|  | **Model 1^1^** | | **Model 2^2^** | | **Model 3^3^** | |
| --- | --- | --- | --- | --- | --- | --- |
|  | β  [95% CI] | p-value | β  [95% CI] | p-value | β  [95% CI] | p-value |
| ***EAAA*** | **1.08**  **[0.96, 1.22]** | **0.187** | **0.96**  **[0.90, 1.03]** | **0.250** | **0.97**  **[0.91,1.02]** | **0.241** |
| *Female* | / |  | / |  | 0.97  [0.93, 1.01] | 0.186 |
| *Age* | / |  | / |  | 1.00  [1.00,1.01] | 0.27 |
| *Married* | / |  | / |  | 1.02  [0.98, 1.06] | 0.273 |
| *Education Status* | / |  | / |  | 0.02***  [1.02, 1.03] | <0.001 |
| N | 1485 |  | 1485 |  | 1475 |  |

^1^Mixed-effect regression with random intercept by healthcare facility (cluster) and a fixed effect for study period, thus assuming a homogeneous secular trend across clusters

^2^ Mixed-effect regression with random intercept by healthcare facility (cluster) and a random slope for study period, thus allowing for varying secular trends across clusters.

^3^ Same as *Model 2* but with additional control variables, including *sex, age, marital status, and education*. All control variables were grand-mean centered.

Supplementary File 1G. OLS Specifications

|  | **Income-generating Time** | | | **Household Living Standards** | | |
| --- | --- | --- | --- | --- | --- | --- |
|  | β  [95% CI] | p_par_ | p_perm_ | β  [95% CI] | p_par_ | p_perm_ |
| ***EAAA*** | 0.02  [-0.36, 0.39] | 0.933 | 0.908 | -0.63  [-1.62, 0.37] | 0.220 | 0.279 |
| *Female* | 0.61***  [0.33-0.90] | <0.001 |  | -0.49  [-1.25, 0.29] | 0.220 |  |
| *Age* | 0.01  [-0.00-0.02] | 0.111 |  | -0.02  [-0.05, 0.01] | 0.316 |  |
| *Married* | 0.37***  [0.12-0.62] | 0.004 |  | 0.35  [-0.31, 1.02] | 0.306 |  |
| *Education Status* | 0.04***  [0.01-0.08] | 0.010 |  | 0.40  [0.31, 0.48] | <0.001 |  |
| N | 3000 |  |  | 1475 |  |  |

*Notes: The estimation is based on the Hussey & Hughes model with control variables.*

Supplementary File 1H. The causal effect of EAAA on a principal component weighted asset and living standard index

|  | **Model 1^1^** | | **Model 2^2^** | | **Model 3^3^** | | **Model 4^4^** | |  |
| --- | --- | --- | --- | --- | --- | --- | --- | --- | --- |
|  | β  [95% CI] | p-value | β  [95% CI] | p-value | β  [95% CI] | p-value | β  [95% CI] | p-value | |
| ***EAAA*** | **-0.15**  **[-0.57, 0.26]** | **0.469** | **-0.15**  **[-0.55, 0.24]** | **0.450** | **-0.16**  **[-0.58, 0.26]** | **0.465** | **-0.15**  **[-0.55, 0.24]** | **0.455** | |
| *Female* | / |  | -0.02  [-0.30, 0.25] | 0.877 | / |  | -0.02  [-0.30, 0.25] | 0.883 | |
| *Age* | / |  | -0.01  [-0.02, 0.00] | 0.290 | / |  | -0.01  [-0.02, 0.01] | 0.283 | |
| *Married* | / |  | 0.01  [-0.23, 0.24] | 0.955 | / |  | 0.01  [-0.23, 0.24] | 0.952 | |
| *Education Status* | / |  | 0.19  [0.16, 0.22] | <0.001 | / |  | 0.19  [0.16, 0.29] | <0.001 | |
| N | 1485 |  |  |  | 1485 |  | 1475 |  | |

Index centered around 0.

^1^Mixed-effect regression with random intercept by healthcare facility (cluster) and a fixed effect for study period, thus assuming a homogeneous secular trend across clusters

^2^Same as *Model 1* but with additional control variables, including *sex, age, marital status, and education*. All control variables were grand-mean centered.

^3^Mixed-effect regression with random intercept by healthcare facility (cluster) and a random slope for study period, thus allowing for varying secular trends across clusters.

^4^Same as *Model 3* but with additional control variables.
